# Prediction of BDS-3 Satellite Clock Bias Based on the Mamba-LSTM Model

**DOI:** 10.3390/s26092643

**Published:** 2026-04-24

**Authors:** Yihao Cai, Hengyi Yue, Tu Yuan, Mengjie Wu

**Affiliations:** Shanghai Astronomical Observatory, Chinese Academy of Sciences, Shanghai 200030, China; cayihao@shao.ac.cn (Y.C.); yhy@shao.ac.cn (H.Y.); yuantu@shao.ac.cn (T.Y.)

**Keywords:** satellite clock bias, multimodal adaptive enhanced model, long short-term memory (LSTM) neural network, BeiDou-3 navigation satellite system (BDS-3)

## Abstract

Since coming into full operation in 2020, the BeiDou-3 Navigation Satellite System (BDS-3) has provided global users with positioning, navigation and time-synchronization services. Satellite clock bias is a key factor that affects real-time precise point positioning (PPP), precise orbit determination and the optimization of navigation message parameters; high-precision prediction of clock bias is therefore critical for improving the accuracy and reliability of BDS-3. To further enhance the prediction accuracy and stability of satellite clock bias, we propose a hybrid model based on Mamba-LSTM. This combined model leverages the strengths of the Multimodal Adaptive Model Building Algorithm (Mamba) and the Long Short-Term Memory neural network (LSTM) to predict satellite clock bias. Using precise BDS-3 satellite clock bias data from the International GNSS Service (IGS), we carried out prediction experiments. First, we compared the proposed model’s predictive performance with that of the Mamba and LSTM models. In short-term (6 h) and long-term (24 h) prediction scenarios, the average prediction RMSE of Mamba-LSTM improved by approximately 41.7% and 48% relative to Mamba, and by approximately 50.4% and 54.7% relative to the LSTM results, respectively. Next, we ran comparison experiments against traditional neural networks—the BP model and the CNN model. In mid-term (12 h) and long-term (24 h) prediction scenarios, the average prediction RMSE of Mamba-LSTM improved by approximately 59.6% and 63.1% compared with BP, and by approximately 52.4% and 56.2% compared with CNN, respectively. The results indicate that the Mamba-LSTM hybrid model can significantly improve the accuracy and stability of satellite clock bias prediction.

## 1. Introduction

Global Navigation Satellite Systems (GNSS) play a pivotal role in modern science and communications, finding extensive application in various high-precision positioning, navigation, and timing (PNT) tasks. Following the global commissioning of the BeiDou-3 Navigation Satellite System (BDS-3), the positioning accuracy and spatiotemporal reference stability it provides have become an indispensable component of the global positioning landscape. Satellite Clock Bias (SCB) is a critical factor affecting navigation system positioning accuracy and time synchronization [1]. SCB represents the timing error caused by the inherent instabilities of satellite atomic clocks; its impact on accuracy becomes particularly pronounced over long-duration measurements. Consequently, high-precision prediction of BDS-3 satellite clock bias is essential for improving both positioning accuracy and the overall reliability of the system. Traditional methods for predicting satellite clock bias have primarily been based on physical models (such as the quadratic polynomial model, QP) [2,3] and statistical approaches (such as the gray system model GM(1,1) [4,5], Kalman filtering [6,7], and the autoregressive integrated moving average model ARIMA [8,9]). However, these methods exhibit significant limitations when dealing with the nonlinear characteristics of BDS-3 satellite clocks, multi-source noise interference, and long-term dependency modeling. For example, physical models rely on prior assumptions about clock physical properties and struggle to adapt to dynamic changes in complex space environments; statistical methods may perform well for short-term predictions but are inefficient for very long sequences and cannot effectively capture nonlinear association patterns [10]. In recent years, with the rise in machine learning—particularly deep learning—an increasing number of studies have adopted data-driven approaches to predict satellite clock bias. The Long Short-Term Memory neural network (LSTM) has demonstrated powerful nonlinear modeling capabilities for time series prediction [11], and LSTM-based models have been widely applied to time series data processing because they are well-suited to capturing long-term dependencies. LSTM can effectively handle the nonlinear and time-varying characteristics present in satellite clock bias data and has therefore achieved promising results in this domain. Nonetheless, when processing very long time series, LSTM still faces issues such as vanishing gradients and information loss, which can lead to suboptimal performance in certain complex scenarios [12].

To address these issues, recent research has gradually begun to introduce hybrid models that combine multiple deep-learning frameworks or integrate other methods to improve prediction accuracy and long-term stability. For example, Li et al. [13] proposed a hybrid model based on LSTM and a self-attention mechanism for GNSS clock bias prediction, achieving favorable predictive performance. By incorporating the self-attention mechanism, the model can better capture global information and improve clock-bias prediction accuracy. Zhao et al. [14] proposed a hybrid model based on a multivariate convolutional neural network (CNN) and a long short-term memory network (LSTM). The CNN is responsible for extracting local spatial features of multi-satellite clock biases (such as inter-satellite correlations), while the LSTM captures long-term temporal dependencies, thus balancing spatial feature extraction and temporal modeling. Experiments show that the CNN–LSTM model outperforms traditional methods in short-, medium-, and long-term prediction. Huang et al. [15] proposed a supervised-learning-based LSTM algorithm for predicting navigation satellite clock bias. A supervised learning mechanism was introduced to guide network training with labeled data (e.g., historical true clock-bias values), enhancing the model’s ability to capture nonlinear features and thereby improving prediction accuracy. Tan et al. [16] proposed a short-term satellite clock-bias prediction method based on complementary ensemble empirical mode decomposition (CEEMD) and a quadratic polynomial model. This method uses CEEMD to decompose the satellite clock-bias time series and extract components at different frequency bands, then fits and predicts each component using a quadratic polynomial model. Experimental results indicate that this method achieves high accuracy and stability in short-term satellite clock-bias prediction.

Despite the promising results achieved in previous studies, current models still struggle with suboptimal prediction accuracy and long-term stability. Specifically, under the complex operational conditions of the BDS-3 satellite system, both traditional physical models and standalone LSTM networks exhibit limited efficacy in clock-bias forecasting. To address these challenges, this paper proposes a novel hybrid architecture, the Mamba-LSTM model, which integrates Mamba [17,18] with an LSTM network for high-precision BDS-3 satellite clock bias prediction. This approach aims to overcome the limitations of existing methods by fusing adaptive sequence modeling with deep-learning-based temporal feature extraction. By employing dynamic selection mechanisms, Mamba can adaptively accommodate varying data characteristics. Furthermore, when synergized with the LSTM network, the proposed hybrid model effectively captures long-term dependencies while maintaining strong adaptability, ultimately delivering superior prediction accuracy and robustness for complex time-series data. To clearly define the prediction task addressed in this paper, we propose a hybrid Mamba-LSTM model for high-precision forecasting of BDS-3 SCB. The model takes as input the preprocessed historical SCB time series, including first-order differencing, gross error detection and correction using the MAD method, and Min-Max normalization. A sliding window strategy is employed to perform epoch-by-epoch prediction, with the prediction horizons mainly set to two scenarios: 12 h and 24 h. The output of the model consists of the predicted satellite clock bias values for each future epoch. All experiments are conducted based on high-precision IGS final clock products, and the performance is evaluated using the root mean square error (RMSE) with respect to the true clock bias values.

Specifically, the main contributions of this paper are as follows:

(1) A novel Mamba-LSTM hybrid model is proposed, which combines the adaptive modeling capability of Mamba with the nonlinear feature extraction ability of LSTM, fully exploring the latent features within the data to improve SCB prediction accuracy.
(2) Experiments conducted on the BDS-3 satellite clock bias dataset demonstrate the superior performance of the proposed model in clock-bias prediction.
(3) Extensive experiments show that the Mamba-LSTM model has strong potential to enhance both the accuracy and stability of BDS-3 satellite clock-bias prediction, providing a new perspective for future research in satellite clock-bias forecasting.

The structure of this paper is arranged as follows: Section 2 introduces the theoretical foundations of the Mamba-LSTM model and its implementation; Section 3 provides a detailed description of the acquisition and preprocessing of the BDS-3 satellite clock bias data; Section 4 presents the prediction results based on the Mamba-LSTM model and their comparison with conventional methods; and finally, Section 5 summarizes the research findings and discusses directions for future work.

## 2. Principles of the Model

### 2.1. Fundamental Principles of the Mamba Model

**Figure 1:**
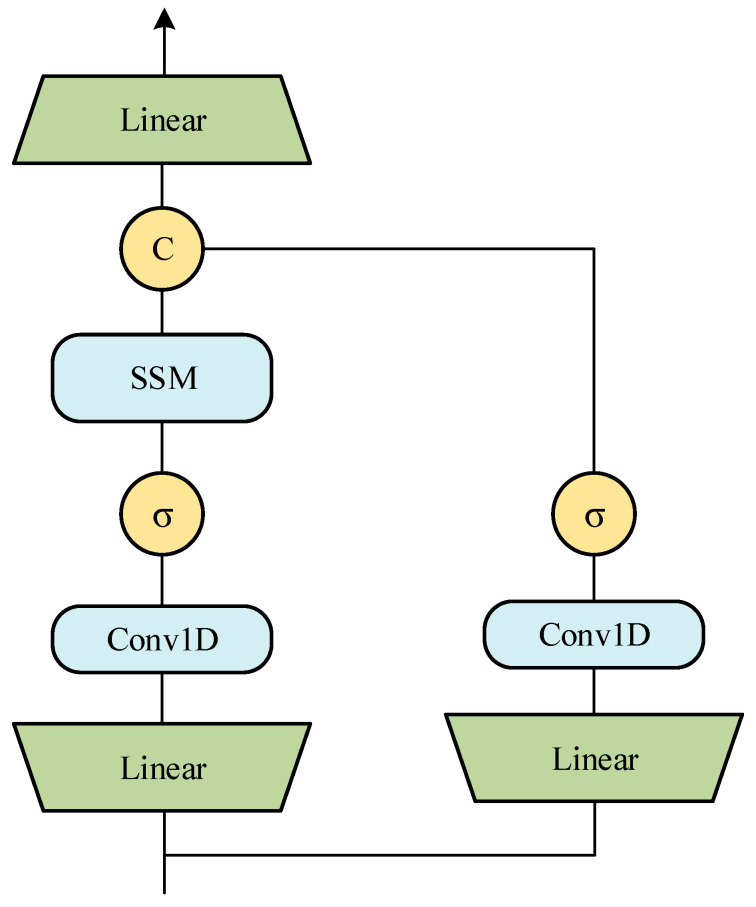
Mamba Block Structure Diagram.

Mamba is a novel sequence modeling architecture, as shown in Figure 1, with its core being the structured state space model (SSM). Unlike Transformer models that rely on self-attention mechanisms with quadratic complexity, Mamba implements an SSM to achieve linear time complexity, making it more efficient for processing long sequences. The basis of Mamba is to map a one-dimensional continuous input signal x(t) through a hidden state h(t) to an output y(t). This process is described by the following linear ordinary differential equation (ODE):

(1) $$\begin{array}{l} h^{\prime }(t)=Ah(t)+Bx(t)\\ y(t)=Ch(t) \end{array}$$

Here, A, B, and C are learnable parameter matrices.

To deploy this continuous-time system on modern computing hardware, it must be discretized. Mamba adopts the zero-order hold rule and introduces a learnable time-scale parameter ∆, converting the continuous parameters A and B into discrete parameters $\overset{–}{A}$ and $\overset{–}{B}$. The discretized state-space system can be expressed as follows:

(2) $$\begin{array}{l} h(t)=\overset{–}{A}h(t-1)+\overset{–}{B}x(t)\\ y(t)=\overset{–}{C}h(t) \end{array}$$

The core innovation of Mamba lies in introducing a selection mechanism. In traditional SSMs, the parameter matrices are fixed and unchanging. However, in Mamba, key parameters (such as B, C, and ∆) are input-dependent. This means the model can dynamically adjust its parameters based on the current input x(t), enabling it to selectively focus on important information in the sequence and filter out irrelevant interference. This selectivity allows Mamba to more effectively compress and process sequence data, demonstrating outstanding performance on various long-sequence modeling tasks.

### 2.2. Fundamental Principles of the LSTM Model

**Figure 2:**
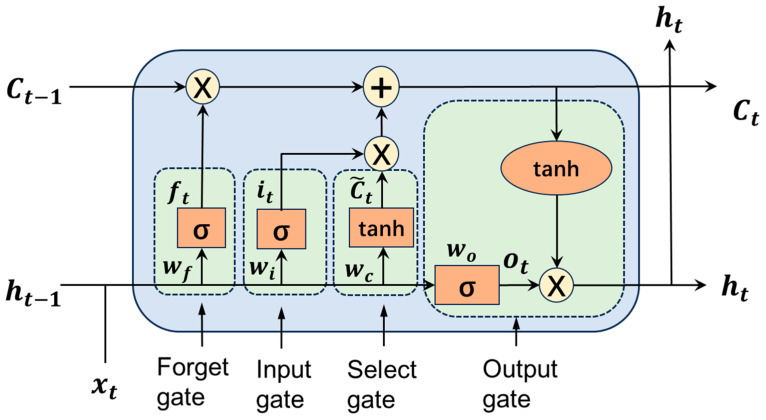
Model structure of the LSTM neural network.

Hochreiter et al. [19] first proposed the LSTM model, which has unique advantages in time series data modeling. LSTM uses a cell to store the long-term state of time series data and consists of three gates: the input gate, forget gate, and output gate. Information is selectively passed at each gate. Figure 2 shows the structure of the LSTM network. The input gate determines how much of the model input will be saved to the cell state, and is implemented through Equation (3).

(3) $$\begin{array}{l} i_{t}=\sigma \left(W_{i}\cdot \left[h_{t-1},x_{t}\right]+b_{i}\right)\\ {\tilde{C}}_{t}=\tanh\left[W_{c}\cdot \left[h_{t-1},x_{t}\right]+b_{c}\right] \end{array}$$

The current time $x_{t}$ and the previous time state $h_{t-1}$ serve as the input gate, then the calculation result is multiplied by the weight matrix, and the update information is determined through the activation function.

The forget gate determines how much of the current model input will be forgotten, and then saves the remaining part to the current cell. The related mathematical expressions are

(4) $$\begin{array}{l} f_{t}=\sigma \left(W_{f}\cdot \left[h_{t-1},x_{t}\right]+b_{f}\right)\\ C_{t}=f_{t}⊙C_{t-1}+i_{t}⊙{\tilde{C}}_{t} \end{array}$$

The forget gate obtains input information from the current time’s input $x_{t}$ and the previous time’s hidden state $h_{t-1}$, and outputs a probability value between 0 and 1. When the probability value is 1, it means retaining all information; when the probability value is 0, it means discarding all information.

The output gate determines what content to output from the current cell state. The related mathematical expressions are

(5) $$\begin{array}{l} o_{t}=\sigma \left(W_{o}\cdot \left[h_{t-1},x_{t}\right]+b_{0}\right)\\ h_{t}=o_{t}⊙\tanh\left(C_{t}\right) \end{array}$$

First, the sigmoid layer determines which part of the cell state needs to be output. Next, the cell state is fed into the “*tanh*” layer, which outputs a probability value between −1 and 1. Finally, this probability value is multiplied by the output result of the sigmoid layer.

In the above equations, W is the weight coefficient matrix, b is the bias vector, and σ and tanh are the sigmoid and tangent activation functions, respectively. Additionally, i, f, C, and o represent the input gate, forget gate, cell state, and output gate, respectively, and ⊙ denotes element-wise matrix multiplication.

### 2.3. Construction of the Mamba-LSTM Model

This paper proposes a hybrid Mamba-LSTM model for the high-precision prediction of BDS-3 SCB. As an adaptive sequence modeling algorithm, Mamba effectively extracts key features across various data dimensions by dynamically adjusting its strategy based on inherent data characteristics. Meanwhile, the LSTM network excels at capturing long-term temporal dependencies. In this integrated framework, Mamba first processes the time-series data to adaptively filter and extract crucial SCB information. Subsequently, these refined features are fed into the LSTM network to model the deep dynamic characteristics and long-term dependencies of the SCB sequence. Consequently, when handling complex SCB signals, the Mamba-LSTM model demonstrates enhanced adaptability and feature extraction capabilities, ultimately yielding significantly more accurate and robust prediction results. The Mamba-LSTM model proposed in this paper aims to synergize the efficient adaptive sequence processing capabilities of Mamba with the powerful nonlinear, long-term dependency modeling of the LSTM network, achieving high-precision prediction of BDS-3 SCB. SCB time series are inherently characterized by complex nonlinearity, time-varying behaviors, and multi-source noise. While an independent LSTM network can effectively capture long-term dependencies, it often struggles when processing ultra-long sequences. Conversely, although Mamba is highly efficient, its nature as a structured state space model (SSM) generally necessitates complementary architectures to fully capture intricate dynamic features. To address these respective limitations, this study integrates Mamba with LSTM, thereby significantly enhancing the hybrid model’s global context awareness and overall predictive performance.

**Figure 3:**
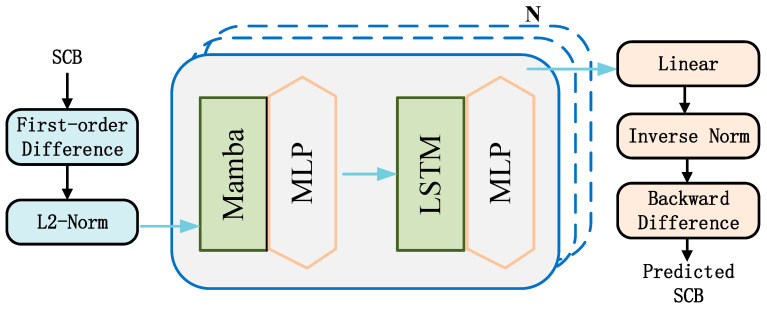
Mamba-LSTM model architecture.

The Mamba-LSTM model we propose adopts an architecture where feature extraction and temporal modeling are connected in series, as shown in Figure 3. First, the preprocessed SCB time series $x=[x_{1},x_{2},\ldots ,x_{T}]\in R^{T}$ is input into the Mamba layer. Mamba utilizes its efficient linear complexity and input-dependent selection mechanism to scan long sequence data.

(6) $$h_{m}=\mathrm{Mamba}(x;\theta_{m})$$

where $\theta_{m}$ denotes a learnable parameter. At this stage, Mamba acts as a powerful adaptive feature extractor, which can selectively retain key information patterns based on the dynamic characteristics of the clock bias data, while filtering out redundant or noisy information, thereby generating a sequence representation rich in key temporal features. Next, the feature sequence extracted by the Mamba layer is used as input and passed to the subsequent LSTM network

(7) $$h_{l}^{\left(t\right)}=\mathrm{LSTM}(h_{m}^{\left(t\right)},h_{l}^{\left(t-1\right)};\theta_{l})$$

As described in Section 2.2, LSTM is very adept at capturing and modeling nonlinear dynamics and long-term dependencies in data through its unique gating mechanisms (input gate, forget gate, output gate). Finally, the hidden state of the LSTM network passes through a fully connected layer (Linear layer) to output the final SCB prediction value

(8) $${\hat{y}}_{t}=Wh_{l}^{\left(T\right)}+b$$

Thus, the complete Mamba-LSTM hybrid model is compactly expressed as follows:

(9) $$\hat{y}=\mathrm{FC}(\mathrm{LSTM}(\mathrm{Mamba}(x)))$$

In this way, the Mamba-LSTM model fully leverages the advantages of both architectures: Mamba is responsible for efficiently and adaptively purifying and compressing long sequence features, while LSTM performs deeper nonlinear and long-term dependency modeling on this basis. The specific parameters of the Mamba-LSTM model are shown in Table 1. This design aims to overcome the limitations of single models, enabling the combined model to predict complex SCB sequences more accurately and robustly.

## 3. Data Processing and Evaluation Methods

### 3.1. Data Preprocessing

The input data for this study comprises the IGS final precise clock products at a 30 s interval. These products are fundamentally estimates derived from the self-consistent adjustment model of the global GNSS network, subject to various influencing factors such as orbit modeling errors, ionospheric and tropospheric delays, and receiver noise. Although the ionosphere-free linear combination utilized by the IGS significantly mitigates first-order ionospheric and plasmaspheric effects, residual higher-order terms can still introduce centimeter-level biases. The preprocessing of the input data primarily involves the following three steps:

First-order differencing: SCB time series inherently exhibit non-stationarity. To enhance sequence smoothness and facilitate the extraction of complex nonlinear features, we apply first-order differencing to the original SCB data. This operation improves data stationarity, which consequently reduces model complexity and enhances overall prediction accuracy [20].

(10) $$\Delta x_{t}=x_{t}-x_{t-1}$$

The sequence used for modeling after differencing is $\Delta x_{t}$.

Gross error detection and repair: Severe gross errors can affect the accuracy of clock bias prediction. The Median Absolute Deviation (MAD) method [21] is used to detect and remove gross errors. The MAD is calculated as follows:

(11) $$MAD=median|x_{t}-\tilde{x}|$$

where $\tilde{x}$ is the median, the threshold is set to 3. If $|x_{t}-\tilde{x}|>3\times \mathrm{MAD}$, the data point is marked as a gross error. For the removed gross errors, cubic spline interpolation can be used to fill them in.

Data normalization: Data normalization can be applied to avoid the impact of different dimensions of feature quantities and target values on prediction performance, accelerate gradient descent during network training, and improve the convenience of model processing. This paper employs Min-Max normalization

(12) $$x^{\prime }=\frac{x-x_{min}}{x_{max}-x_{min}}$$

Map $x^{\prime }$ to the interval [0, 1].

### 3.2. Network Model Training and Prediction

To ensure the reliability and generalization capability of the model, all datasets in this study are univariate time series consisting solely of SCB values; therefore, no class imbalance issue exists. Accordingly, a time-series-specific data splitting strategy is adopted, as follows:

Training set: the complete data from the first day (2880 epochs) is used for model parameter optimization;

Validation set: the last 20% of the training data are selected in chronological order for hyperparameter tuning;

Test set: the completely independent data from the following day (2880 epochs) are used for final performance evaluation.

**Figure 4:**
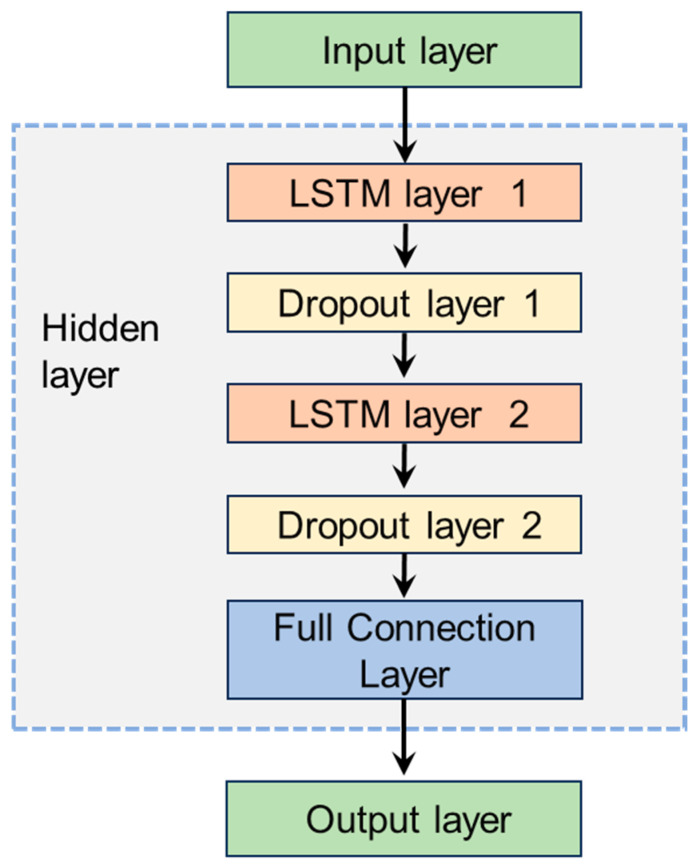
LSTM model design.

**Figure 5:**
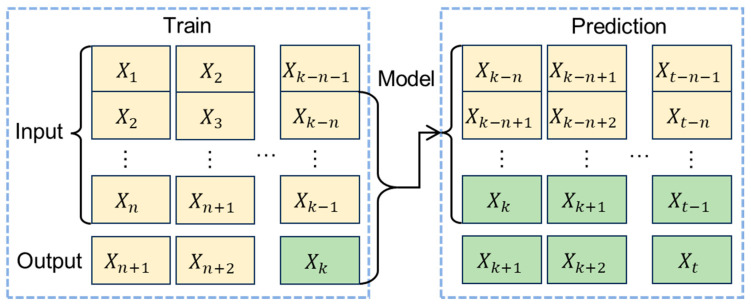
Training and prediction of data.

Model Structure Design and Parameter Settings: The LSTM model designed in this paper consists of an input layer, hidden layers, and an output layer. The number of neurons in the input layer equals the number of input data points. The hidden layers consist of 2 LSTM layers, each connected to a dropout layer containing 32 hidden nodes. The dropout layer, during the training process, has a dropout rate of 0.2 to prevent overfitting. Figure 4 shows the specific LSTM model framework design. Table 2 describes the specific parameter settings of the LSTM model. This paper adopts a sliding window approach for sample generation and prediction. The window size is 60, and the slide step is 1, as shown in Figure 5.

### 3.3. Data Post-Processing

After completing the LSTM model prediction, the predicted values are obtained and then subjected to denormalization and inverse first-order differencing to obtain the final predicted SCB sequence. The experimental results and analysis will be detailed in the next section.

### 3.4. Evaluation Methodology

This paper utilizes the post-processed precise clock offset products provided by IGS as our data source to ensure the quality and reliability of the experimental data. This data source is widely used globally due to its high precision, and it also guarantees the credibility of our model’s performance. The experimental design in this paper is as follows: using the data from the previous day (20 July 2025) for training, and employing the trained model to predict the SCB data for the next day (21 July 2025). The data time interval is 30 s, covering a total of 5760 epochs.

To deeply analyze and evaluate the model’s predictive performance, we compared the actual SCB data provided by IGS with the model’s predicted values. In this evaluation, we use the root mean square error (RMSE) as the metric to assess prediction accuracy.

(13) $$RMSE=\sqrt{\frac{1}{n}\sum_{i=1}^{n}{\left(y_{i}-{\hat{y}}_{i}\right)}^{2}}$$

where ${\hat{y}}_{i}$ represents the clock bias of the i-th epoch predicted by the model, $y_{i}$ represents the actual clock bias of the i-th epoch provided by IGS, and n represents the total number of predicted epochs.

## 4. Experiments and Analysis

### 4.1. Model Performance Analysis

To comprehensively evaluate the predictive performance of our proposed model on SCB, we selected representative BDS-3 satellites equipped with different types of atomic clocks for experimental verification, specifically C24 (rubidium atomic clock), and C30 and C46 (hydrogen atomic clocks). Incorporating these diverse satellite types allows for a robust assessment of the model’s reliability and applicability. Furthermore, we conducted independent comparative experiments utilizing the standalone LSTM, Mamba, and our hybrid Mamba-LSTM models to demonstrate the superiority of the proposed approach. For the training phase, we utilized one full day of data (comprising 2880 epochs) to optimize the network, which was subsequently employed to forecast the SCB for the following day. To maintain temporal continuity, data from the final sliding window of the training period served as the initial input for the prediction phase. Specifically, we focused on 6 h and 24 h forecasting scenarios to rigorously assess the model’s predictive capabilities. Table 3 shows the root mean square error (RMSE) of SCB predictions for 6 h and 24 h under different models. From the table, it is easy to conclude that, in the 6 h and 24 h prediction scenarios, the average prediction RMSE of Mamba-LSTM improved by approximately 41.7% and 48% compared to Mamba, respectively, and the average prediction RMSE of Mamba-LSTM improved by approximately 50.4% and 54.7% compared to LSTM results, respectively. We observe that the prediction accuracy of the Mamba-LSTM model is significantly better than that of the LSTM model and the Mamba model, and the improvement in prediction accuracy is more pronounced in the 24 h prediction scenario. This trend has been verified in C24 (rubidium atomic clock), C30 (hydrogen atomic clock), and C46 (hydrogen atomic clock). This result indicates and highlights the stability and superiority of the prediction performance of the model in this paper.

To evaluate the effect of training dataset size on the performance of the Mamba-LSTM model, datasets spanning 1 day, 3 days, and 5 days were employed as training samples, respectively. In this experiment, the model’s objective was to predict the SCB data for 21 July 2025. The detailed results are presented in Table 4. As can be observed from the table, the forecasting results do not exhibit significant improvement as the training dataset size increases, and the overall prediction accuracy remains at a comparable level. Based on these experimental observations, it can be inferred that while a larger training dataset may introduce more features, it may simultaneously incorporate more noise, thereby preventing any substantial gain in model performance. Therefore, it can be concluded that a training dataset of 1 day is sufficient to yield a well-performing predictive model. Accordingly, taking computational resource constraints into consideration, a dataset size of 1 day is adopted as the training sample for the model in this study.

### 4.2. Comparative Experimental Analysis

To rigorously evaluate the predictive performance of the proposed Mamba-LSTM model, we established a comparative experimental framework incorporating Backpropagation (BP) neural networks and Convolutional Neural Networks (CNNs) as baselines. The BP network was selected due to its robust nonlinear mapping capabilities, which are widely recognized in time-series forecasting [22,23,24]. Similarly, CNNs have been effectively applied to various time-series tasks [25,26], utilizing multiple convolutional layers to progressively extract abstract, high-level temporal features. By benchmarking against these established architectures, we aim to objectively assess the advantages of our proposed hybrid model in the specific domain of SCB prediction.

**Figure 6:**
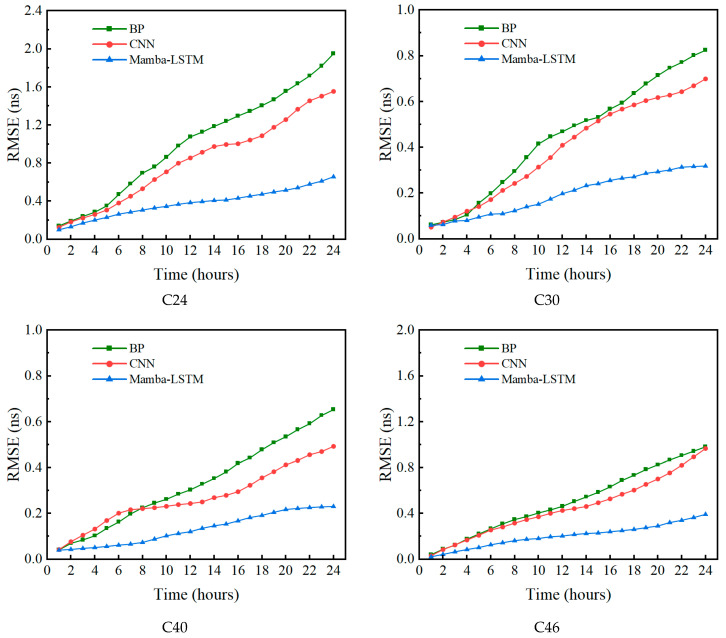
RMSE variation over time for the three models.

To verify the applicability and robustness of the proposed approach, experiments were conducted using data from four distinct types of BDS-3 satellites. We utilized one day’s data as the training dataset to forecast the SCB for the subsequent 12 h and 24 h horizons. This experimental design provides a thorough understanding of model performance across varying prediction lengths, offering valuable insights for future SCB research. Finally, Figure 6 compares the temporal evolution of the prediction accuracy among the three models, displaying the Root Mean Square Error (RMSE) trajectories for the four different satellite types as the forecasting horizon extends.

From Figure 6, it can be observed that in both short-term and long-term prediction scenarios, the prediction accuracy of the Mamba-LSTM model is superior to that of the BP and CNN models, especially as the prediction time extends, the prediction accuracy of the Mamba-LSTM model changes less, and the prediction accuracy is much higher than the other two models, which indicates that its prediction accuracy and stability are both higher than the other two models. To compare the prediction performance of the three models in detail, we focus on analyzing the specific performance of these three models in the 12 h and 24 h prediction tasks. From Table 5 and Table 6, it can be seen that in the medium-term (12 h) and long-term (24 h) prediction scenarios, the average prediction RMSE of Mamba-LSTM improved by approximately 59.6% and 63.1% compared to BP, respectively, and the average prediction RMSE of Mamba-LSTM improved by approximately 52.4% and 56.2% compared to CNN results, respectively. This indicates that as the prediction duration increases, the Mamba-LSTM method is significantly superior to the other two methods in terms of prediction accuracy and stability, and has a significant advantage in controlling the accumulation of prediction errors over prediction time.

### 4.3. BDS-3 Full Satellite Experiment Analysis

To further assess the stability and overall performance of the proposed Mamba-LSTM model, comprehensive evaluations were conducted across all available BDS-3 satellites within the dataset. For each satellite, we utilized one day’s data to train the model, subsequently forecasting the SCB for the following 12 h and 24 h horizons. Consistent with our previous analyses, the predictive performance was rigorously benchmarked by comparing the RMSE values of the Mamba-LSTM model against those of the BP and CNN baselines.

**Figure 7:**
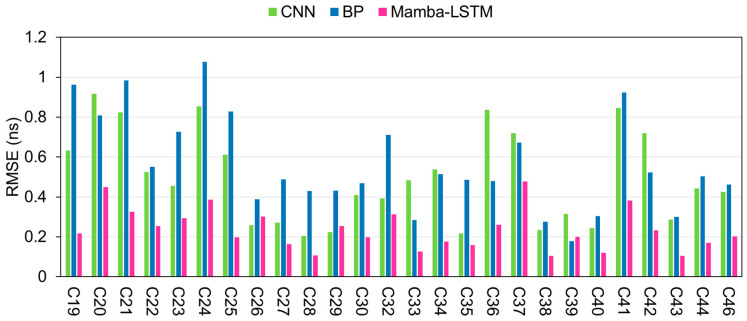
The prediction RMSE of the three models for all BDS-3 satellites within a 12 h forecast period.

**Figure 8:**
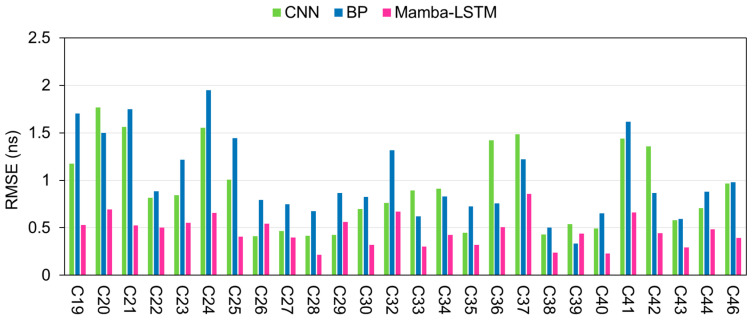
The prediction RMSE of the three models for all BDS-3 satellites within a 24 h forecast period.

Figure 7 and Figure 8 show the predicted RMSE values of the three models for all BDS-3 satellites within the 12 h and 24 h prediction durations. From the figures, it can be seen that, compared to the BP and CNN models, the Mamba-LSTM integrated model always achieves the lowest predicted RMSE values for all satellites, which fully highlights the outstanding performance of the Mamba-LSTM integrated model in high-precision SCB prediction. This also demonstrates the strong reliability and superiority of the Mamba-LSTM integrated model in SCB prediction.

To further validate the effectiveness of our proposed method, we expanded the experimental dataset to include clock deviation data spanning a full week, specifically from 21 July 2025 to 27 July 2025. Table 7 presents the weekly average RMSE statistics for the BP, CNN, and Mamba-LSTM models across 12 h and 24 h forecast durations. As shown in the table, the proposed Mamba-LSTM model consistently outperforms the other two baseline models in both scenarios. In the 12 h forecasting scenario, the Mamba-LSTM model achieves the lowest average RMSE of 0.2529 ns, which is significantly lower than that of the BP model (0.5867 ns) and the CNN model (0.4438 ns). As the prediction duration extends to 24 h, although the prediction errors of all models inevitably accumulate, the Mamba-LSTM model still maintains the highest accuracy. Its average RMSE at 24 h is 0.4953 ns, representing a substantial improvement over the BP model (1.0925 ns) and the CNN model (0.8075 ns). These statistical results firmly demonstrate the superior predictive accuracy and robust long-term stability of the Mamba-LSTM architecture.

## 5. Conclusions and Future Direction

Focusing on the BDS-3 satellite clock bias (SCB) time series, this study proposes a novel Mamba-LSTM hybrid model. By integrating the distinct strengths of both the Mamba architecture and the LSTM network, our approach enables high-precision SCB prediction. Extensive comparative experiments and analyses demonstrate that the proposed Mamba-LSTM method significantly enhances both the accuracy and stability of clock bias forecasting. In summary, our method exhibits the following key advantages: Compared with single models (such as Mamba and LSTM methods), the Mamba-LSTM combined model shows significant improvements in the stability and accuracy of predicting satellite clock bias.

The prediction errors of traditional neural network methods (such as BP and CNN methods) increase rapidly with the extension of prediction time, while the Mamba-LSTM method has a significant advantage in controlling the accumulation of prediction errors over prediction time, making it more suitable for medium- and long-term predictions.

The Mamba model and LSTM network are effective tools for processing time series data. For typical time series data processing problems, we combined the advantages of both, achieved effective application in satellite clock bias prediction, and obtained good results. This work makes a beneficial attempt at in-depth research on satellite clock bias prediction problems and provides new ideas for further research in this field.

The method proposed in this paper still has some aspects that can be further studied and improved.

The proposed method can be further studied for fusion with other methods to further improve its prediction performance.

The model’s computational complexity is slightly higher than that of a single LSTM. It is necessary to further study optimization methods for hyperparameter selection and training to further enhance the computational efficiency of this method.

Space weather phenomena (such as solar activity cycles and magnetic storms) can significantly alter the thermal environment of the ionosphere and satellites. During intense magnetic storms, a decline in Precision Orbit Determination (POD) accuracy and unaccounted-for higher-order ionospheric delays often manifest as high-frequency noise or sudden anomalies in IGS apparent clock deviation estimates. Its robustness under extreme space weather disturbances (such as strong magnetic storms) has not yet been fully verified; future work could further systematically investigate the impact of strong magnetic storms on the model.

## Figures and Tables

**Table 1:** Mamba-LSTM model parameter list.

No.	Parameter	Value
1	Mamba layer	1
2	State dimension	64
3	LSTM layers	2
4	Hidden units	32
5	Output layer	1
6	Activation functions	tanh

**Table 2:** LSTM model parameter list.

No.	Parameter	Value
1	Optimizer	Adam
2	Loss function	MSE
3	Number of neural network layers	2
4	Training times	100
5	Batch quantity	512
6	Learning rate	0.01

**Table 3:** RMSE over 6 h and 24 h for different models.

PRN	Models	RMSE in 6 h (ns)	RMSE in 24 h (ns)
C24	LSTM	0.5176	1.3574
	Mamba	0.4379	1.1738
	Mamba-LSTM	0.2645	0.6572
C30	LSTM	0.2299	0.7579
	Mamba	0.2020	0.6935
	Mamba-LSTM	0.1085	0.3183
C46	LSTM	0.2513	0.8626
	Mamba	0.2087	0.7255
	Mamba-LSTM	0.1269	0.3925

**Table 4:** The RMSE over 24 h for different training datasets.

PRN	1 Day Data (ns)	3 Days Data (ns)	5 Days Data (ns)
C24	0.6572	0.6045	0.5839
C30	0.3183	0.3691	0.3073
C46	0.3925	0.4166	0.4037

**Table 5:** Accuracy statistics of 12 h forecasts of the three models.

PRN	BP	CNN	Mamba-LSTM
	RMSE (ns)	RMSE (ns)	RMSE (ns)
C24	1.0765	0.8545	0.3854
C30	0.4685	0.4093	0.1977
C40	0.3035	0.2439	0.1205
C46	0.4619	0.4261	0.2028

**Table 6:** Accuracy statistics of 24 h forecasts of the three models.

PRN	BP	CNN	Mamba-LSTM
	RMSE (ns)	RMSE (ns)	RMSE (ns)
C24	1.9501	1.5537	0.6572
C30	0.8246	0.6996	0.3183
C40	0.6533	0.4927	0.2306
C46	0.9813	0.9667	0.3925

**Table 7:** Weekly average statistics predicted by the three models.

Forecast Duration	BP	CNN	Mamba-LSTM
	RMSE (ns)	RMSE (ns)	RMSE (ns)
12 h	0.5867	0.4438	0.2529
24 h	1.0925	0.8075	0.4953

## Data Availability

The experimental data in the manuscript are all public data and can be downloaded from https://cddis.nasa.gov/archive/gnss/products/2364/ (accessed on 22 April 2026).
